# Immune and Stroma Related Genes in Breast Cancer: A Comprehensive Analysis of Tumor Microenvironment Based on the Cancer Genome Atlas (TCGA) Database

**DOI:** 10.3389/fmed.2020.00064

**Published:** 2020-03-05

**Authors:** Ming Xu, Yu Li, Wenhui Li, Qiuyang Zhao, Qiulei Zhang, Kehao Le, Ziwei Huang, Pengfei Yi

**Affiliations:** Department of Breast and Thyroid Surgery, Union Hospital, Tongji Medical College, Huazhong University of Science and Technology, Wuhan, China

**Keywords:** breast cancer, tumor microenvironment, immune infiltration, prognosis, ESTIMATE algorithm, The Genome Cancer Atlas database

## Abstract

**Background:** Tumor microenvironment is essential for breast cancer progression and metastasis. Our study sets out to examine the genes affecting stromal and immune infiltration in breast cancer progression and prognosis.

**Materials and Methods:** This work provides an approach for quantifying stromal and immune scores by using ESTIMATE algorithm based on gene expression matrix of breast cancer patients in TCGA database. We found differentially expressed genes (DEGs) through limma R package. Functional enrichments were accessed through Gene Ontology (GO) analysis and Kyoto Encyclopedia of Genes and Genomes (KEGG) pathway analysis. Besides, we constructed a protein-protein network, identified several hub genes in Cytoscape, and discovered functionally similar genes in GeneMANIA. Hub genes were validated with prognostic data by Kaplan-Meier analysis both in The Cancer Genome Atlas (TCGA) database and Molecular Taxonomy of Breast Cancer International Consortium (METABRIC) database and a meta-analysis of hub genes prognosis data was utilized in multiple databases. Furthermore, their relationship with infiltrating immune cells was evaluated by Tumor IMmune Estimation Resource (TIMER) web tool. Cox regression was utilized for overall survival (OS) and recurrence-free survival (RFS) in TCGA database and OS in METABRIC database in order to evaluate the impact of stromal and immune scores on patients prognosis.

**Results:** One thousand and eighty-five breast cancer patients were investigated and 480 differentiated expressed genes (DEGs) were found based on the analysis of mRNA expression profiles. Functional analysis of DEGs revealed their potential functions in immune response and extracellular interaction. Protein-protein interaction network gave evidence of 10 hub genes. Some of the hub genes could be used as predictive markers for patients prognosis. In this study, we found that tumor purity and specific immune cells infiltration varied in response to hub genes expression. The multivariate cox regression highlighted the fact that immune score played a detrimental role in overall survival (HR = 0.45, 95% CI: 0.27–0.74, *p* = 0.002) and recurrence-free survival (HR = 0.41, 95% CI: 0.22–0.77, *p* = 0.006) in TCGA database. These result was confirmed in METABRIC database that immune score was a protector of OS (HR = 0.88, 95% CI: 0.77–0.99, *p* = 0.039).

**Conclusions:** Our findings promote a better understanding of the potential genes behind the regulation of tumor microenvironment and cells infiltration. Immune score should be considered as a prognostic factor for patients' survival.

## Introduction

Over the past few years, tumor microenvironment has been one of the fastest developing and most promising fields in breast cancer research. An increasing number of studies have investigated that immune microenvironment not only modulates immunotherapy but also promotes prognosis of patients with breast cancer (1–3). To estimate the stromal and immune infiltration level of tumor and provide clues for researches on this field, Estimation of STromal and Immune cells in MAlignant Tumor tissues using Expression data (ESTIMATE) (4) is modeled and tested to calculate tumor purity, stromal and immune scores of patients via expression profile and give an overall view of tumor microenvironment. As indicated in recent work, high or intermediate immune score of breast cancer could bring better disease-free survival or overall survival (5). Other than confirming this discovery, we intended to investigate the pathways and genes that potentially affect tumor infiltration.

There are some works concerning characteristic genes that could impact tumor cellularity. Gene modifiers such as point mutations or deletions on several growth factor receptors, pattern recognition receptors, transcription factors, and apoptosis-related proteins are summarized to be associated with nonmalignant tumor microenvironment in breast cancer and affects most aspects of breast cancer biology such as tumorigenesis, progression, and metastases (6). The amount of somatic copy-number variation (SCNV) of immune genes are negatively related to immune signatures but positively related to stroma infiltration in the analysis of lung adenocarcinoma (7). Moreover, chemokines, interleukins, interferons, and their receptors are differentially expressed in different phenotypes of breast cancer with varied infiltration levels of microenvironment cells (8). On the basis of these researches, we attempted to identify pivotal genes contributed to tumor infiltration in breast cancer. Since multiple kinds of cells including tumor infiltrating lymphocytes (TILs), dendritic cells, tumor-associated macrophages (TAM), and tumor-associated neutrophils (TAN) are all related to tumor treatment and prognosis inspected in several different works (9–13), we also predicted these genes' relationships with immune cell aggregation and tumor prognosis.

## Materials and Methods

### Data Collection and Preparation

The Cancer Genome Atlas-Breast Cancer (TCGA-BRCA) RNAseqV2 gene expression data and clinical data were obtained from the TCGA Data Portal (14). Altogether, 1,096 female breast cancers from TCGA with normalized gene expression and specific clinical status were collected and analyzed. Transcriptional values were Log2-transformed from the normalized fragments per kilobase transcript per million mapped reads values using R package “limma” in R 3.6.0.

We accessed Molecular Taxonomy of Breast Cancer International Consortium (METABRIC) (15) on cBioportal website (16, 17) for gene expression and clinical data. One thousand, four hundred twenty-four samples with specific genes expression and complete clinical and prognostic data were chosen and downloaded for further analysis.

### ESTIMATE Algorithm and Identification of Stromal and Immune Groups

ESTIMATE algorithm (Estimation of STromal and Immune cells in MAlignant Tumor tissues using Expression data) (4) was applied to value stromal and immune microenvironment infiltration based on gene expression data. The analysis method is integrated in “estimate” R package in R 3.6.0. In ESTIMATE algorithm, the expression profiles of two independent sets of 141 genes are considered to represent the extent of tumor stromal and immune infiltrations. Thus, we extracted these expression matrixes from RNASeqV2 data to calculate stromal and immune scores in TCGA-BRCA samples. ESTIMATE score, which is the summation of stromal and immune score from individual case, is defined as tumor purity. To explore the possible association between stromal and immune score and clinical statistics, characteristics such as age at diagnosis, ER status, PR status, HER2 status, histological type, menopause status, PAM50 subtypes, pathologic T, N, M, and stage were evaluated. Unpaired *t*-test was used to compare stromal and immune scores between young (≤55 years old) and old patients (>55 years old) while one-way analysis of variants and Least Significant Difference (LSD) test were used to carry out the significances of other characteristics. Since indeterminate data showed biased outcomes, we omitted them while *post hoc* was conducted.

Medians of stromal and immune scores were considered as cutoffs for high and low score group demarcation. The medians we used here were 531.15 in stromal group and 617.78 in immune group, respectively.

### Identification of Valid Differentially Expressed Genes (DEGs) and Their Functional Analysis

Differentially expressed genes (DEGs), defined as dysregulated genes with |logFC| > 1 and FDR < 0.05 between high and low score groups in this study, were operated and clustered via R package “limma.”

To explore pivotal genes' function in tumor microenvironment infiltration, we intended to find genes mediated both in stromal and in immune compartment. Therefore, DEGs in stromal and immune groups were overlapped and genes were selected only when they changed synchronously in both groups (i.e., genes were upregulated or downregulated in both groups). Validated DEGs were visualized through Venn graphs and a heatmap via R package pheatmap.

### Functional Enrichment, Pathway Analysis, and PPI Network of Differentially Expressed Genes (DEGs)

Using the list of DEGs above, Gene Ontology (GO) analysis and Kyoto Encyclopedia of Genes and Genomes (KEGG) pathway analysis were conducted on WebGestalt.org (18). The R package “ggplot2” was used to visualize the results of GO analysis and KEGG pathway with enrichment *p* < 0.05 and FDR < 0.1. The top 15 pathways with highest enrichment scores were shown in biological processes (BPs) enrichment since more than 15 pathways were found during the analysis.

As we know, ESTIMATE algorithm uses 141 genes separately to calculate stromal and immune scores, in order to rule out the impact of these genes on functional network enrichment, we prudently excluded them if they are in DEGs list. After that, other valid DEGs were carried out for further protein-protein interaction (PPI) network construction. Setting minimum required interaction score as high confidence (0.7), PPI network was constructed on STRING website to elucidate potential interactions between DEGs. MCODE application (19) in Cytoscape software (20) was utilized to extract network modules and identify hub genes if they showed strong connections in the network. GeneMANIA website (21) was used to predict the relationship between hub genes and their functionally similar genes. Interaction network was downloaded and rearranged according to their interplays.

### Predictive Value of Hub Genes in Survival Analysis

Hub genes were unraveled from protein-protein interactions. All samples' clinical and survival data were reanalyzed from BRCA database and the expression related prognosis were validated in METABRIC database. We divided patients into different groups by the medians of hub genes expression. Kaplan-Meier analysis was performed for survival curves and the significance was determined by log-rank, during which R packages survival and survminer in R 3.6.0 were used to analyze and sketch Kaplan-Meier curves.

### Meta-analysis of Hub Genes' Survival in Organized Public Databases

We downloaded survival data with cox regression of all hub genes in PROGNOSCAN website (22), recently updated in 2019 April. In total 19 databases were included and used for meta-analysis, 18 of which are Gene Expression Omnibus (GEO) databases and one is ArrayExpress database. The hazard ratios (HRs) of every hub gene were reorganized. Summarized HRs were illustrated using R package forest plot. Overall survival (OS) in five databases, disease free survival (DFS) in 12 databases, disease-specific survival (DSS) in three databases and disease metastasis free survival (DMFS) in nine databases were all evaluated.

### Hub Genes' Correlation With Immune Cells Infiltration

To explore the relations between hub genes and the infiltrating immune cells, we utilized TIMER (Tumor IMmune Estimation Resource) web tool (23, 24) to calculate coefficients of correlation between hub genes expression and infiltrated immune cells including B cells, CD4^+^ T cells, CD8^+^ T cells, macrophages, neutrophils, and dendritic cells. The correlation heatmap was drawn using coefficients and *p*-values extracted from TIMER calculation via pheatmap R package.

### Cox Regression and Survival Analysis

Complete clinical, pathological and prognostic data of 1,085 breast cancer patients were obtainable for further analysis. All ESTIMATE results were reorganized with clinical and pathological characteristics for subsequent statistical analysis. Quantitative stromal score and immune score were categorized into high and low groups taking medians as cutoffs. Age at diagnosis was defined as young (≤55 years) and old (>55 years). Univariate and multivariate cox regression were used to shed light on the relationship between clinical patterns and the immune microenvironment in TCGA and METABRIC database. Hazard ratios and corresponding confident intervals were calculated through R package survival using survival data.

## Results

### Stromal/Immune Scores Were Distributed Diversely in Terms of Clinical Characteristics

**Figure 1:**
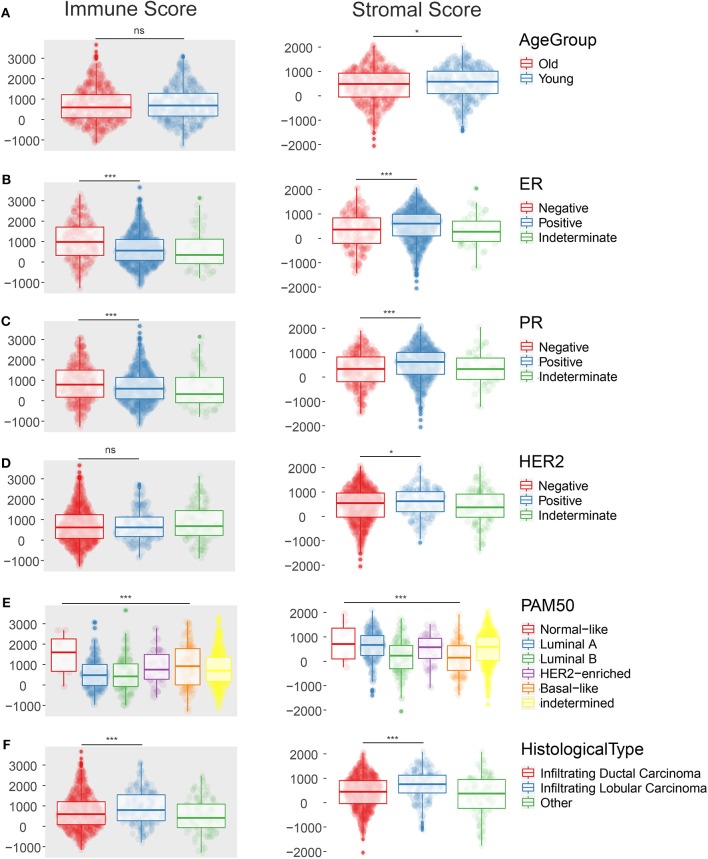
The scatter plot shows that the immune score and the stromal score are distributed differently among clinical characteristics such as Age at diagnosis **(A)**, ER status **(B)**, PR status **(C)**, HER-2 status **(D)**, PAM50 subtypes **(E)**, Histological type **(F)**. *p < 0.05; ***p < 0.001; ns, not significant.

ESTIMATE algorithm gave stromal and immune scores for all 1,096 samples, among which 1,085 cases with clinical data were used. We firstly compared different distribution of these scores with respect to clinical characteristics as follows: age at diagnosis, ER status, PR status, HER2 status, histological type, menopause status, PAM50 subtypes, pathologic T, N, M, and tumor stage. Indeterminate data were omitted while each specific factor was inspected and analyzed as shown in Figure 1. Among all the characteristics displayed in Supplementary Table S1, we found the immune infiltration score was diversely distributed in terms of age at diagnosis, ER status, PR status, HER2 status, histological type, and PAM50 subtypes. Young patients (≤55 years old) showed elevated stromal scores (*p* = 0.02) but similar immune scores (*p* = 0.18) compared to old patients. Only 517 cases were defined in PAM50 subtypes. Luminal B subtype and basal-like subtype were associated with lower stromal score (*p* < 0.001) while luminal A and B subtype were less infiltrated by immune cells (*p* < 0.001). Invasive lobular carcinoma had higher level of stromal score (*p* < 0.001) but lower infiltration of immune cells (*p* < 0.001) compared with invasive ductal carcinoma, revealing the possible differences in tumor microenvironment during tumorigenesis in IDC and ILC. Positive ER and positive PR status showed same trend on lower stromal infiltration (*p* < 0.001) and higher immune infiltration (*p* < 0.001). Interestingly, positive HER2 status was only related to lower stromal score (*p* = 0.036) but not significantly relevant to immune score (*p* = 0.705).

As for other clinical features such as menopause status, pathologic T, N, M, and tumor stage, only stromal score was significantly associated with some terms, such as pathologic T (*p* = 0.003), pathologic N (*p* = 0.003), and tumor stage (*p* = 0.058) when indeterminate data were excluded.

### Up- and Down-Regulated Genes in Stromal and Immune Groups Were Overlapped to Generate Valid Differentially Expressed Genes (DEGs)

In order to evaluate the possible impact of stromal and immune scores on breast cancer, we investigated the expression patterns in different stromal and immune groups. DEGs were compared between high- and low-score stromal or immune groups with |logFC| < 1 and *p* < 0.05 through R package “limma.” Total of 1,075 upregulated genes and 212 downregulated genes were found in different stromal groups. Meantime, 1,251 upregulated genes and 187 down regulated genes were identified in different immune groups.

**Figure 2:**
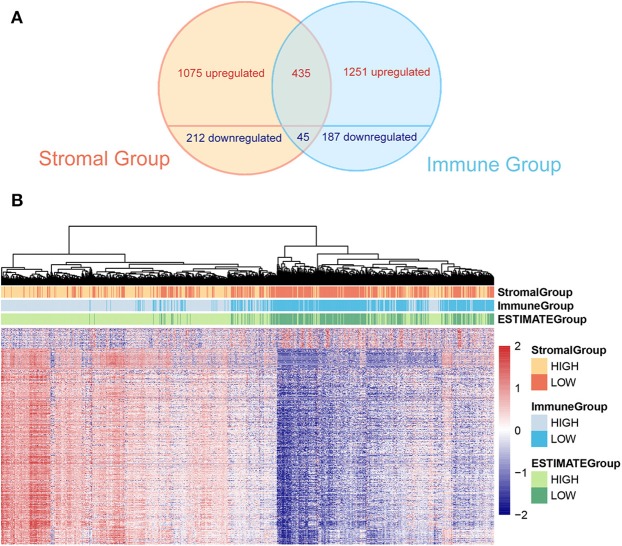
**(A)** The Venn graph shows intersection of DEGs in stromal and immune group. Four hundred and thirty-five upregulated and 45 downregulated genes are found. **(B)** The heatmap demonstrates DEGs and the stromal score, the immune score, and the tumor purity (ESTIMATE score) at the top of the heatmap.

To minimize the systematic error from group classification, the overlap of genes with same trends in both stromal and immune groups were considered as valid DEGs. Consequently, 435 overexpressed genes and 45 underexpressed genes were found (the overlap of valid DEGs was shown in Venn graph in Figure 2A). The heatmap of gene expression and the stromal, immune and ESTIMATE scores of 1,096 patients were shown and clustered in Figure 2B. The original expression data was uploaded in Supplementary Materials. From the clustering, we could find out that dysregulated genes showed a similar expression trend along with stromal, immune and ESTIMATE scores, indicating that they expressed coordinately and probably cooperated among certain biological processes.

### Gene Ontology (GO) and Kyoto Encyclopedia of Genes and Genomes (KEGG) Analysis Showed Functional Enrichment in Immune Regulations

**Figure 3:**
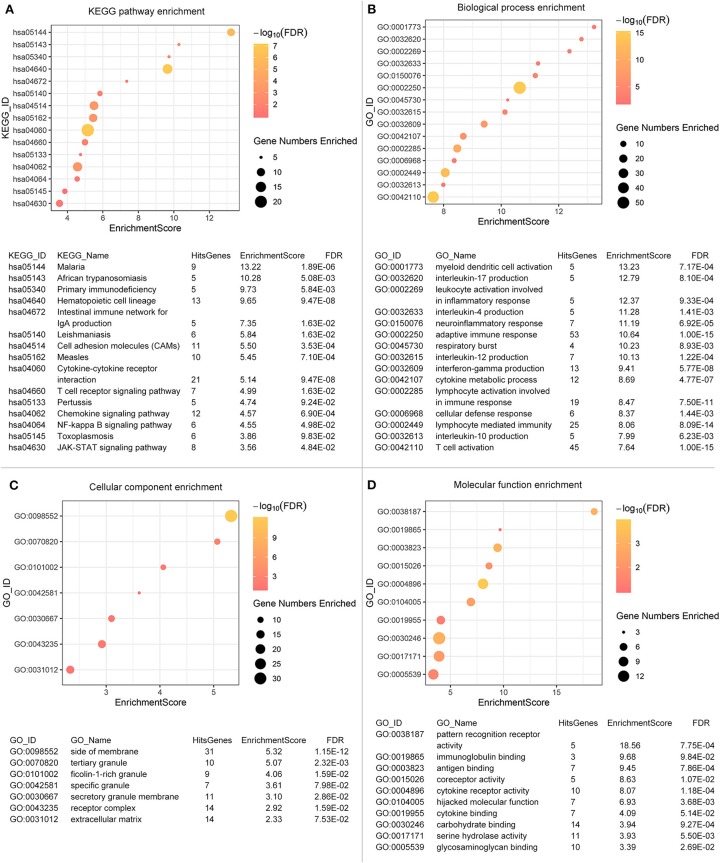
Functional enrichments are operated. Fifteen biological processes **(A)**, ten molecular functions **(B)**, seven cellular components **(C)** and fifteen KEGG pathways **(D)** are illustrated. The color of the dots demonstrates -Log10 (FDR). Therefore, yellow dots shows greater FDR than red ones. The size of the dots indicates the number of genes enriched in the analysis.

To explore the underlying interplay of these valid DEGs, GO analysis, and KEGG pathway enrichments were performed. As shown in Figure 3A, 15 biological processes (BPs) were enriched, such as immune cells activation, immune response, and cytokine metabolic processes. Ten molecular functions (MFs) including glycose metabolism, antigen, and immunoglobulin binding were found to be related as shown in Figure 3B. Seven cellular components (CCs) regarding extracellular and receptor complex, granule generation and secretion were found as shown in Figure 3C.

Regarding to the KEGG pathway analysis as shown in Figure 3D, infectious diseases related pathways were enriched such as malaria, leishmaniasis and measles. Besides, NF-κB signaling pathway and JAK-STAT signaling pathway were also enriched, which revealed potential mechanisms and pathways activated during tumor progression. Immune processes and regulations were significantly enriched in both GO and KEGG analysis. Even though we excluded genes used for scoring, we could not deny the possibility that the enrichment result was influenced partially by scoring process. The GO identifiers/KEGG identifiers, enrichment scores and False Discovery Rates (FDRs) of the enrichment analysis was displayed below the graph.

### Hub Genes Were Extracted From Protein-Protein Interaction (PPI) Network and Validated in Different Databases

**Figure 4:**
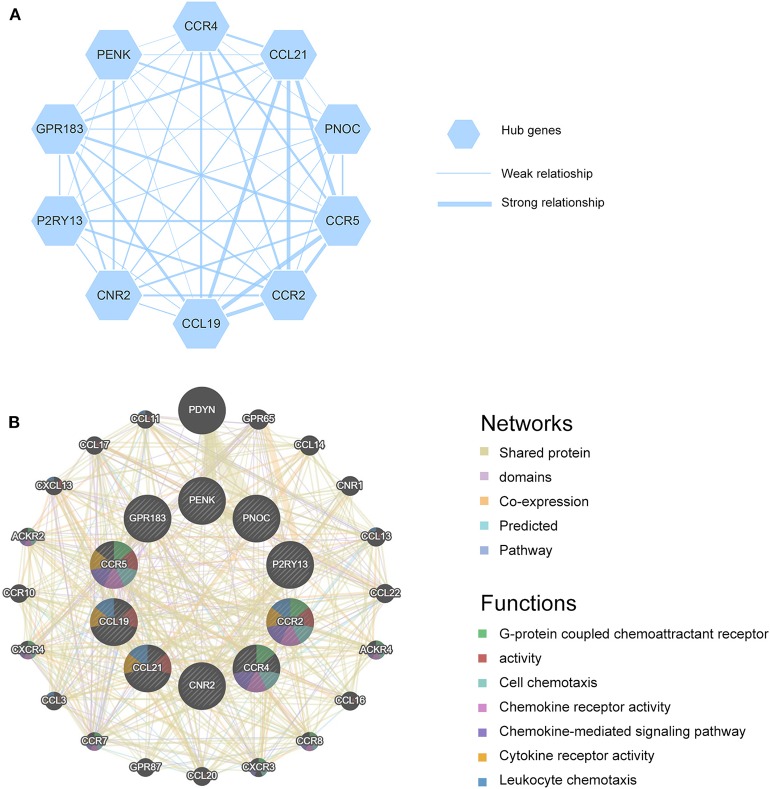
**(A)** Hub genes are shown and the thickness of the line indicates the extent of the relationship between two genes. **(B)** GeneMANIA is used to identify predicted genes correlating with hub genes, 20 of the predicted genes are located in the outer circle while hub genes are drawn in the inner circle. The color of the line illustrates different type of their relationships. The color inside the gene dots indicates functions which these genes are involved in.

All valid DEGs except genes employed in ESTIMATE algorithm were used to predict PPI network in STRING website. Setting minimum required interaction score as high confidence (0.7), 191 genes were found and analyzed in STRING. All genes were mutually connected and interacted, constructing a sketch of the interplay network. Further analysis was conducted in Cytoscape and 10 genes were screened out as hub genes (CCR4, CCL21, PNOC, CCR5, CCR2, CCL19, CNR2, P2RY13, GPR183, PENK) and a potential interaction sketch map was shown in Figure 4A. We also uploaded hub genes to GeneMANIA website to predict functionally similar genes. As a result, twenty genes emerged as shown in Figure 4B, among which 17 genes had shared protein domains with hub genes, 12 genes were co-expressed with hub genes, 8 genes were co-localized with hub genes, 10 genes shared similar pathways with hub genes. We also visualized predicted functions in the network. Hub genes were arranged at the inner circle while predicted genes at the outer circle. Immune response and chemokine related functions were enriched, such as G-protein coupled chemoattractant receptor activity, cell chemotaxis, chemokine receptor activity, chemokine-mediated signaling pathway, cytokine receptor activity, leukocyte chemotaxis, and chemokine receptor binding. These results strongly supported the hypothesis that hub genes were interacted with each other and played important roles in immune processes during the tumor progression.

**Figure 5:**
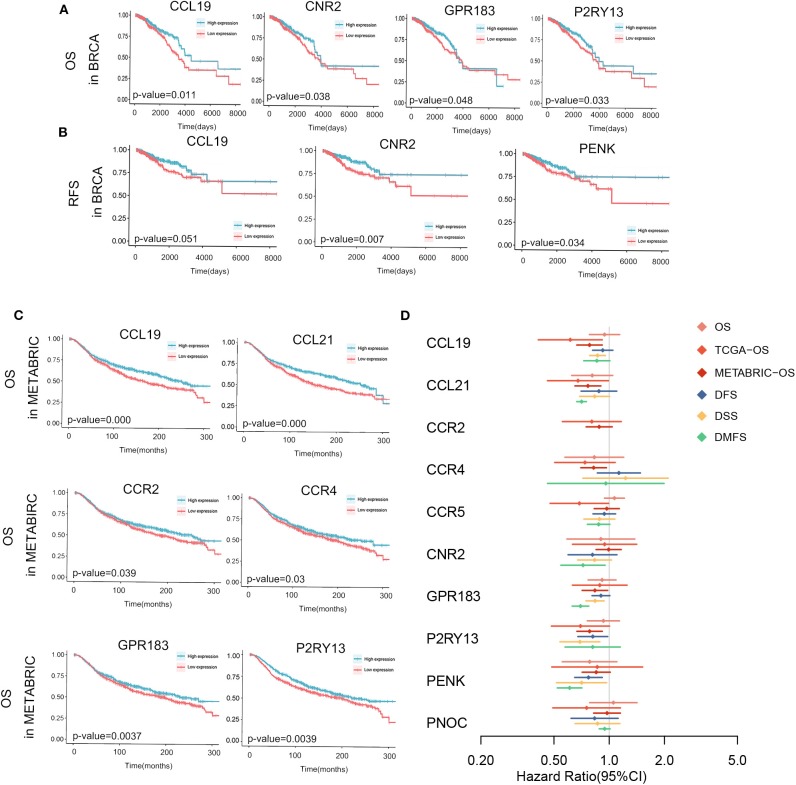
Kaplan-Meier survival analyses and meta-analysis of survivals in multiple databases are shown. Significant OS **(A)**, RFS **(B)** in TCGA-Breast Cancer and OS of METABRIC **(C)** are sketched and *p*-values are in the plots. **(D)** Meta-analysis of the survival with endpoints as OS, DFS, DSS, and DMFS and cox regression of OS in TCGA and METABRIC of 10 hub genes. *P* < 0.05 is used as significant criteria. DFS, disease free survival; DMFS, disease metastasis free survival; DSS, disease specific survival; OS, overall survival.

Moreover, we investigated these genes in TCGA database, METABRIC database and GEO databases to find their potentials as prognostic factors. In TCGA database, higher expression of CCL19, CNR2, P2RY13 and GPR183 showed significantly higher OS rate and longer median survival time with *p* < 0.05 in Figure 5A and Supplementary Figure S1. And high expression of CCL19, CNR2, and PENK showed prolonged RFS in Figure 5B and Supplementary Figure S1. In METABRIC database, we applied Kaplan-Meier analysis of overall survivals. The relevant high expression of CCL19, CCL21, CCR2, CCR4, GRP183, or P2RY13 significantly extended patients' lifespan as shown in Figure 5C and Supplementary Figure S1.

All information about 19 databases were attached in Supplementary Table S2. The results of meta-analysis in multiple databases of 10 hub genes and the cox regression of genes in TCGA database and METABRIC database were together demonstrated in Figure 5D and Supplementary Figure S2. CCR2 was not in the meta-analysis since it was not evaluated in any of the multiple databases but its relationship with prognosis in TCGA and METABRIC database was demonstrated. As obviously indicated, all genes tended to be protective in patients survivals. As we can see, all genes were not significant in meta-analysis of OS. The reason of this phenomenon could be the limited follow-up time and small sample size. Other than the results in meta-analysis, the genes which were possibly influence OS included CCL19, CCL21, CCR4, GPR183, and P2RY13. In all hub genes, P2RY13 and PENK played a significantly beneficial role in DFS as CCL19, GPR183, P2RY13, PENK in DMFS, and CCL21, CNR2, GPR183, PENK in DSS.

Overall, CCL19, CCL21, GPR183, P2RY13, and PENK were potential protective factors in breast cancer. These genes as prognostic biomarkers in breast cancer need systematic and thorough researches in the future.

### The Correlation Between Hub Genes and Immune Infiltration Was Identified

**Figure 6:**
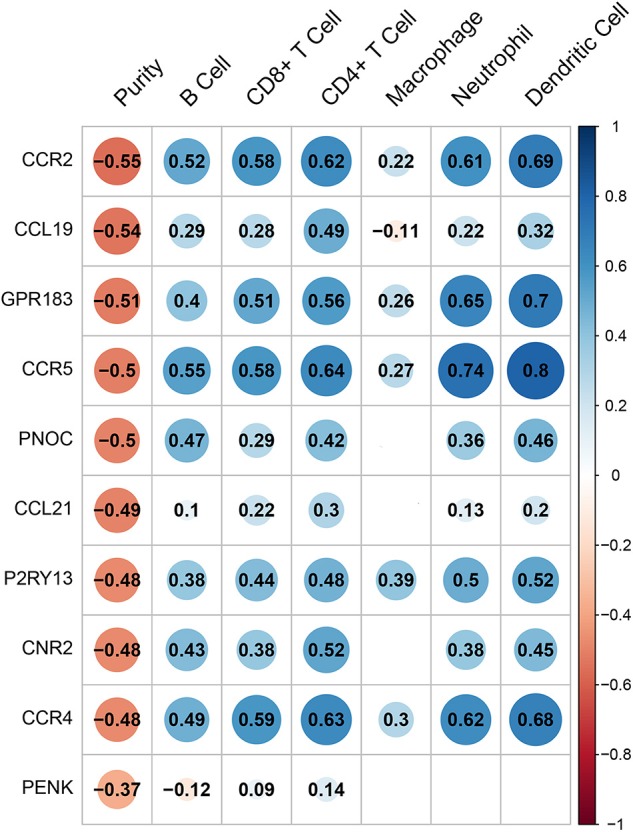
The correlation heatmap demonstrates the relationship between gene expressions and immune cells infiltration. Dots size shows the extent of their relationships and dots color indicates if they are positive-related (blue dots) or negative-related (red dots). The number inside dots indicates the coefficients of correlation between genes expression and cells infiltration. Blank squares mean insignificance (*p* > 0.05).

Hub genes expression was analyzed by TIMER (Tumor IMmune Estimation Resource) web tool in order to predict their probable influences on immune cells infiltration including lymphoid cells such as B cells, CD4^+^ T cells, CD8^+^ T cells and myeloid cells including macrophages, neutrophils, and dendritic cells. The scatter diagrams and fitting curves generated by TIMER were attached in Supplementary Figure S3. Immune cells infiltration was generally linked to hub genes expression as shown in Figure 6. All genes were associated with tumor purity (coefficient above 0.37). Most of the genes were related to certain types of immune cells. However, we could easily tell that CCL21 and PENK have very limited correlation with immune cell infiltration while CCR2, GPR183, CCR5, and CCR4 showed a strong correlation with all immune cells except macrophages. GPR183 and CCR5 were slightly more relevant to myeloid cells infiltration while other genes showed strong mediating functions in both lymphoid and myeloid cells. On the other aspect, macrophages were mildly affected by CCR2, GPR183, CCR5, and CCR4 while P2RY13 showed the most remarkable correlation with macrophages (coefficient = 0.39).

### Cox Regression With Matrix and Immune Compartment and Clinical Characteristics

**Figure 7:**
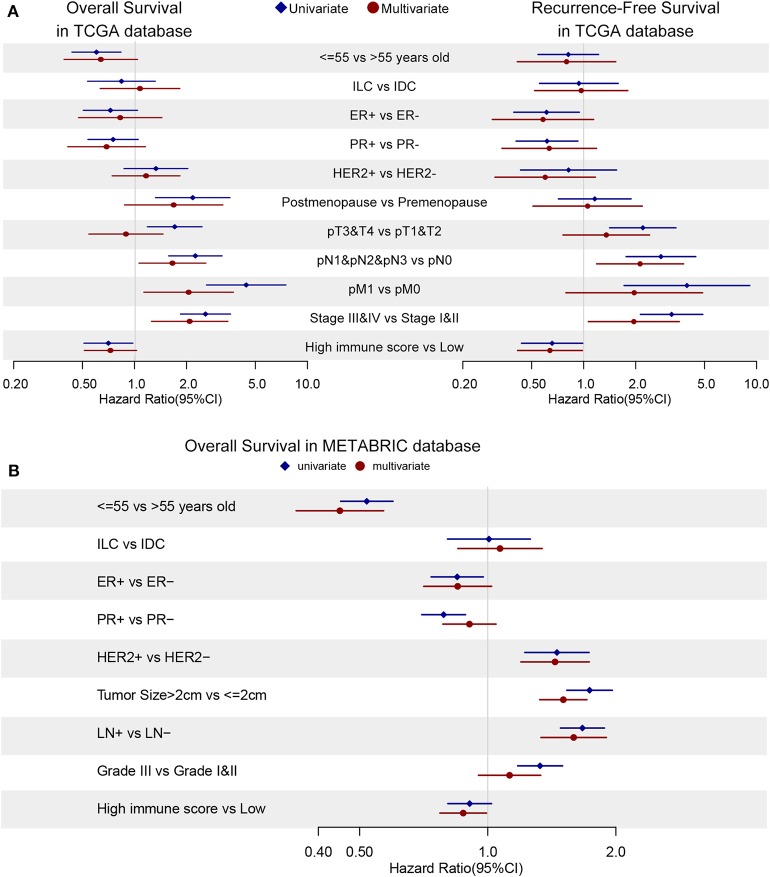
The cox regression is applied to discover hazard ratios (HRs) of clinical characteristics and immune scores in OS (left) and RFS (right) in TCGA database **(A)** and OS in METABRIC database **(B)**. Blue diamonds and red dots demonstrates univariate and multivariate analysis, respectively. The confidence intervals are shown as the length of the line. Lines cross HR = 1.0 indicates their insignificance.

Cox regression was used to evaluate the relationship between risk factors and survival time. Univariate and multivariate cox regression were conducted using R language and forest plots were demonstrated in Figure 7A. In univariate analysis, young patients (≤55 years old) (HR = 0.60, 95% CI: 0.43–0.83, *p* = 0.002), post-menopause (HR = 2.16, 95% CI: 1.31–3.54, *p* = 0.002, compared to pre-menopause), T3&T4 (HR = 1.70, 95% CI: 1.18–2.44, *p* = 0.004, compared to T1&T2), N1&N2&M3 (HR = 2.24, 95% CI: 1.57–3.19, *p* < 0.001, compared to N0), M1 (HR = 4.41, 95% CI: 2.6–7.48, *p* < 0.001), stage III & IV (HR = 2.56, 95% CI: 1.83–3.57, *p* < 0.001, compared to stage I & II), high immune scores (HR = 0.70, 95% CI: 0.51–0.97, *p* = 0.031) were prognostic factors. Based on the univariate analysis, we include immune score in next multivariate analysis but stromal score and ESTIMATE score were excluded since these scores were linearly dependent on each other as shown in Supplementary Figure S4. In multivariate analysis for overall survival, young patients (≤55 years old) (HR = 0.58, 95%CI: 0.35–0.95, *p* = 0.032), N0 (HR = 0.57, 95% CI: 0.37–0.88, *p* = 0.012), M0 (HR = 0.48, 95% CI: 0.25–0.84, *p* = 0.011), stage I & II (HR = 0.52, 95% CI: 0.31–0.88, *p* = 0.014), high immune scores (HR = 0.45, 95% CI: 0.27–0.74, *p* = 0.002) turned out to be protective factors.

As for recurrence-free survival (RFS), only N0 (HR = 0.45, 95% CI: 0.25–0.81, *p* = 0.007), stage I & II (HR = 0.54, 95% CI: 0.29–0.99, *p* = 0.048) and high immune score (HR = 0.41, 95% CI: 0.22–0.77, *p* = 0.006) showed their potential protective effects in multivariate cox regression (Figure 7A). In univariate analysis, reduced hazard ratios on recurrence-free survival could be observed in positive ER status (HR = 0.61, 95% CI: 0.39–0.94, *p* = 0.026), positive PR status (HR = 0.61, 95% CI: 0.41–0.93, *p* = 0.020), T1 (HR = 0.45, 95% CI: 0.29–0.71, *p* < 0.001) and M0 (HR = 0.25, 95% CI: 0.11–0.58, *p* = 0.001) patients group.

The univariate and multivariate cox regression of overall survival were applied in METABRIC database and immune score was confirmed to be a protector (HR = 0.88, 95% CI: 0.77–0.99, *p* = 0.039) with younger age (HR = 0.45, 95% CI: 0.35–0.57, *p* = 0.039), ER-positive (HR = 0.85, 95% CI: 0.71–1.02, *p* = 0.08), HER2-negative (HR = 0.69, 95% CI: 0.58–0.84, *p* < 0.001), smaller tumor size (HR = 0.66, 95% CI: 0.58–0.75, *p* < 0.001) and no metastasis lymph nodes (HR = 0.63, 95% CI: 0.53–0.75, *p* < 0.001) as demonstrated in Figure 7B. Cox regression results could be checked in Supplementary Tables S3, S4.

## Discussion

Tumor microenvironment in breast cancer has arisen concentration these years. On the basis of the fact that tumor microenvironment has manifested its influence on diagnosis, classification, treatment and prognosis, all carcinoma types are divided into different subtypes by immune genes and immune cell infiltration in several models. Pan-cancer immune analysis uses expression signatures for clustering and six immune subtypes of cancer in spite of primary sites are generated (25). The six subtypes possess distinctive characteristics on infiltrated immune cells, somatic variation, immunomodulators and prognosis. Similar analysis is applied in triple negative breast cancer that 29 immune signatures are used as criteria for clustering and defining immunity levels of high, intermediate and low (26). In a recent work, 72-gene test panel are drawn from more than 2,000 cases and tested in public and private databases for subtyping and immunity-adjusted risk of distant metastasis analysis in breast cancer (27). These findings are all focused on clustering patients into different subtypes using immunity-related genes and cells but give little evidence on the possible mechanism of these genes or cells in tumor microenvironment.

Based on these studies, we calculated tumor purity, stromal, and immune infiltration levels based on mRNA expression. From the description of clinical characteristics and prognosis, there was a significantly positive correlation between the majority of clinical features and stromal scores but immune scores. It might suggest the independent impact of immune infiltration on prognosis and survival. The result of the cox regression was coordinated with the hypothesis. Immune score was a significant protector no matter in univariate or multivariate cox analysis. Interestingly, high immune score was found to be associated with ER negative and PR negative status while the latter two are considered as factors for poor prognosis in breast cancer stratification and treatment. The connection between immune infiltration and ER status could be seen as well in ductal carcinoma *in situ* among women patients (28, 29). However, there is necessity that the level of immune or stromal infiltration should be detected further using flow cytometry and/or single-cell sequencing and their relationships with clinical features can be restated.

Furthermore, we bioinformatically investigated possible pathways and hub genes triggered or mediated the variation of tumor cellularity. Immune response and extracellular interactions were found during the enrichment analysis. Cell membrane activity and granule generation in CC, cytokines, membrane receptor binding and activities in MF, immune cell activation, interleukins production, immune responses in BP all combined to sketch a picture of immunity processes in tumor progression. More importantly, there are two signaling pathways highlighted in KEGG enrichment other than immunity-centered pathways, which are Janus kinase/signal transducer and activator of transcription (JAK/STAT) and NF-κB pathway. The JAK/STAT pathway is recognized as a downstream of a variety of cytokines, hormones, and growth factors. This rapid membrane-to-nucleus signaling plays an significant role both in metabolism and in immune process (30–32). NF-κB pathway, activated by extracellular molecules, is also a convincing induction of tumorigenesis and metastasis (33, 34). These two pathways interact with each other and modulate immune cells in tumors (35–37). Our results have given evidence of their critical roles in tumor microenvironment.

Among DEGs, 10 hub genes (CCR4, CCL21, PNOC, CCR5, CCR2, CCL19, CNR2, P2RY13, GPR183, PENK) were found and their prognostic effects and relationship with infiltrated cells were predicted. The chemokines CCL19 and CCL21 were two of C-C chemokine ligands that shared the same chemokine receptor CCR7. Researchers have found that CCR7 is highly expressed on human B cells, expanded T cells instead of naïve T cells (38) and dendritic cells (39) and its expression on peripheral T cells was positively related to the chemotactic migration of T cells to CCL19 and CCL21 (40). On the other hand, CCR7 mediated chemotaxis required the existence of CCL19 or CCL21 (41). *In vitro* experiment indicated that recombinant CCL19 showed potent chemotactic activity for T-cells and B-cells but not for granulocytes and monocytes. Combined with our analysis in Figure 6, CCL19 showed a chemotactic role in B cells, T cells, and dendritic cells. In contrast, recombinant murine CCL21 was tested to be chemotactic for activated T cells, but not for B cells, macrophages, or neutrophils *in vitro* (42). CCL19 significantly enhanced breast cancer patients' prognosis in our work and we speculated it could work in accordance with these immune cells infiltration. In our analysis, CCL21 expression was positively correlated with T cells and dendritic cells, less correlated to B cells, neutrophils but not related to macrophages infiltration. Researchers have found that CCL21/CCR7 chemokine axis not only induced lymphangiogenesis in breast cancer (43), but also promoted breast cancer cells migration and metastasis (44). We could further explain whether and how immune cells modulate these processes experimentally.

Chemokines receptors CCR2, CCR4, and CCR5 were extremely associated with gathering of immune cells except macrophages as in Figure 6. These genes are all functional receptors with several cognate chemokines and mediates chemotaxis and migration of immune cells through intracellular signaling pathways. However, these genes effect on breast cancer should be interpreted with caution since they mainly expressed in leukocytes. In Yang et al.'s work (27) on breast cancer, blood single-cell RNA-seq was used to exclude active genes in the blood and they precisely located genes that were activated in tumor tissues. More confirmation like this can be applied to chemokine receptors because the expression change of these genes can be resulted from the infiltration of leukocytes. GPR183, P2RY13 and CNR2 are all G-protein coupled receptors, in which GPR183 and P2RY13 are not only expressed in immune cells but also in breast tissues while CNR2 mostly expressed in immune organs. Their effect in immune responses and cell chemotaxis have been discovered. GPR183 mediated in immune response, intestinal immunity and inflammation (45) when activated in T cells, B cells, dendritic cells and macrophages (46–48). In glioblastoma multiforme, GPR183 contributed to chemotactic migration of THP-1 cells toward tumor (49). P2RY13, in another hand, was analyzed in lung adenocarcinoma and associated with survival of patients (50), which was similar to our analysis. But if this effect could be explained by high immune infiltration needs to be further investigated. We found P2RY13 as an promoter of high proportion of immune cells in tumor, on the contrary, P2RY13 was negatively correlated with acute inflammatory score in Crohn's disease (51). Due to different pathogenesis of Crohn's disease and breast cancer, we need to interpret the result with caution. Even though CNR2 expressed high only in the immune system, it was unraveled to enhance head and neck squamous carcinoma progression (52) and impaired prostate cancer cell migration by heterodimerized with CXCR4 (53). These genes function are not illustrated clearly, but their prognostic effect in breast cancer should be interpreted with deeper experiments.

PENK and PNOC, precursor proteins of enkephalin and nociceptin, respectively, are highly expressed genes in the central neuron system as transmitters to opioid receptor and commonly work in pain transmission and perception. However, the promoter hypermethylation of PENK has been indicated in several malignancies including bladder cancer (54, 55), colorectal cancer (56) and pancreatic cells (57, 58). In researches, PENK has been reported to be required for apoptosis induction in response to activation or overexpression of p53 and p65 through NF kappa B signaling pathway (59), which is a pathway that is activated in inflammation and enriched in our functional analysis. PNOC has merely been focused in cancer but it is assumed to express in immune system. Its expression could be down-regulated by LPS or IL-10 in human whole blood cultures (60). *In vivo* studies in mice and/or rats revealed the interaction between nociceptin and several inflammatory mediators in immune system such as TNF-α, IFN-γ, and IL-1β (61–63).

To further investigate these genes relationship with immune cells, we predicted gene-immune cell interactions. It is reasonable that higher expression of hub genes is correlated with lower tumor purity and higher immune cells infiltration. Numerous studies have attempted to summarize the suppressive functions of immune infiltration through immune cells, matrix cells (64) and immune-vascular crosstalk (65). Existing researches have recognize the critical role of mononuclear immune cells including CD4^+^ T cells, CD8^+^ T cells, macrophages, and natural killer cells (9, 66–70). In non-small cell lung cancer (NSCLC), lymphocytes especially T and B plasma cells were significantly associated with better OS (71). These results also corroborate the idea in renal cell carcinomas that different types of tumor-infiltrating immune cells modulated in different subtypes of renal carcinomas and prolonged OS or DFS (28). However, a contradictory study indicated cancer-associated fibroblasts activation correlated with tumor-associated macrophages Infiltration and lymph node metastasis could promote tumor progression by mediating inflammatory reactions in triple negative breast cancers (29). Besides, stromal cells are found to act as crucial factors during chemotherapy and endocrine therapy in breast cancer (72–74). In our study, different genes had diverse correlation coefficients with different immune cells, presenting the unique function of genes in immune infiltration and tumor-immune interplay. This may suggest the immune infiltration possibly slows down tumor growth and metastases through its specific ways. But *in vitro* and *in vivo* experiments are needed to confirm their functions and interplays.

Current understanding of tumor microenvironment has showed that high immune score, indicating highly active immune cells infiltrations inside solid tumors, brings prognostic benefits including highly differentiated phenotype (75), less switch to invasive carcinoma (76), higher pathological complete response rate to neoadjuvant treatment (77) and better OS and DFS (9, 78). These results are in concordant with our analysis that immune score is a protective factor in breast cancer prognosis.

Our findings may help to understand the phenomena of stromal and immune infiltration in breast cancers. Since our analysis is totally based on bioinformatic methods, the results should be interpreted cautiously. We could only offer a relation that the hub genes and functional pathways could work in the process of tumor microenvironment and affect the prognosis of breast cancer patients, but we have very restricted evidence of their potential causal relationship. Some of our hub genes (CCL19, CCL21, CCR2, CCR4, CCR5, CNR2) are mainly expressed in immune cells or organs, resulting in the possibility that their expressions are mostly affected by immune cells infiltration. But there is still necessity to identify their potential to predict prognosis and if possible, their function in tumor infiltration. Multi-color flow cytometry has already been used in breast cancer immunophenotyping (79) and tumor microenvironment (80). It could measure the proportion of several kinds of immune cells and sort those we are interested for *in vivo* and *in vitro* experiments. Subsequently we could clarify the specific effects of diverse immune cells to promote or prohibit tumor progression. Meanwhile, with the development of single-cell sequencing, diverse immune phenotypes in breast cancer has been illuminated (81). A combined analysis of eight patients indicated the variability of immune cells in different patients and the work also proposed the phenomenon of continuous activation in T cells in breast tumor. Abundant questions raised for further promotion on determining the interaction and crosstalk between genes and tumor-infiltrated cells in experiments.

## Data Availability Statement

The datasets generated for this study can be found in the TCGA Data Portal https://portal.gdc.cancer.gov/ for the Cancer Genome Atlas-Breast Cancer and the cBioPortal website https://www.cbioportal.org/ for Molecular Taxonomy of Breast Cancer International Consortium (METABRIC).

## Author Contributions

PY and YL proposed the topic. MX, YL, QZhao, and QZhan contributed to the design and interpretation of the study. YL, MX, WL, KL, and ZH conducted the calculation and figures in R. All authors discussed the results and contributed to the final manuscript.

### Conflict of Interest

The authors declare that the research was conducted in the absence of any commercial or financial relationships that could be construed as a potential conflict of interest.
