# Supplementary material for: Immune and Stroma Related Genes in Breast Cancer: A Comprehensive Analysis of Tumor Microenvironment Based on the Cancer Genome Atlas (TCGA) Database
Source: Front Med (Lausanne). 2020 Mar 5;7:64. doi: 10.3389/fmed.2020.00064 (PMC7066229; doi:10.3389/fmed.2020.00064)
Supplement: Supplementary file 1 [file Table_1.docx]

Supplementary Materials

**Supplementary Table S1. Stromal/immune scores are distributed in terms of clinical characteristics**

|  | No.of patients | Stromal Score |  | Immune Score |  |
| --- | --- | --- | --- | --- | --- |
|  |  | (Mean±SEM) | ***p*-value** | (Mean±SEM) | ***p*-value** |
| **Total** | 1085 | 457.8±20.8 |  | 723.7±25.8 |  |
| **Age** |  |  | **0.02** |  | 0.18 |
| ≤55 years old | 470 | 510.3±30.3 |  | 763.7±39,.1 |  |
| ＞55 years old | 615 | 417.7±28.3 |  | 693.2±34.2 |  |
| **PAM50** |  |  | **<0.001** |  | **<0.001** |
| Normal-like | 8 | 776.2±287.6 |  | 1487.2±362.4 |  |
| Luminal A | 231 | 615.5±40.5 |  | 536.6±47.7 |  |
| Luminal B | 122 | 183.1±63.1 |  | 499.2±76.3 |  |
| HER2-enriched | 58 | 486.9±79.7 |  | 839.6±106.7 |  |
| Basal-like | 98 | 127.9±68.2 |  | 953.4±104.6 |  |
| Indeterminate | 568 | 502.2±28.6 |  | 785.8±35.3 |  |
| **Histologic Type** |  |  | **<0.001** |  | **<0.001** |
| Invasive Ductal Carcinoma | 774 | 413.9±23.9 |  | 701±30.7 |  |
| Invasive Lobular Carcinoma | 204 | 716.8±42.4 |  | 913.9±57.5 |  |
| Indeterminate | 107 | 281.9±79.6 |  | 525.5±80.6 |  |
| **ER** |  |  | **<0.001** |  | **<0.001** |
| Positive | 238 | 309.4±46.4 |  | 1027.2±61.4 |  |
| Negative | 797 | 511.6±23.7 |  | 639.9±28 |  |
| Indeterminate | 50 | 306.9±93.1 |  | 615.3±137.6 |  |
| **PR** |  |  | **<0.001** |  | **0.003** |
| Positive | 342 | 307±37.7 |  | 853.5±50 |  |
| Negative | 690 | 542.6±25.3 |  | 666.5±30.2 |  |
| Indeterminate | 53 | 327.2±92.8 |  | 631.2±136.4 |  |
| **HER2** |  |  | **0.037** |  | 0.364 |
| Positive | 773 | 445.5±25.3 |  | 706.1±31.2 |  |
| Negative | 193 | 561±41.7 |  | 732±55.1 |  |
| Indeterminate | 119 | 370.2±64.1 |  | 824.6±80.1 |  |
| **Menopause Status** |  |  | 0.097 |  | 0.755 |
| Pre | 229 | 524.4±41.7 |  | 720.5±54.3 |  |
| Peri | 40 | 603.2±107.9 |  | 688.7±119.4 |  |
| Post | 703 | 442.4±26.1 |  | 738.9±32.5 |  |
| Indeterminate | 113 | 367.2±69.1 |  | 647.9±83.2 |  |
| **Pathologic T stage** |  |  | **0.003** |  | 0.09 |
| T1 | 279 | 576.5±39.9 |  | 773.2±54.2 |  |
| T2 | 627 | 419.3±27.6 |  | 726.3±33.8 |  |
| T3 | 137 | 438.9±55.3 |  | 700.1±68.5 |  |
| T4 | 39 | 361±116.4 |  | 486.6±98.3 |  |
| Indeterminate | 3 | -413.3±112.9 |  | -253.5±187.1 |  |
| **Pathologic N stage** |  |  | **0.003** |  | **0.024** |
| N0 | 510 | 413.5±31.6 |  | 754.5±40.1 |  |
| N1 | 359 | 472.8±34.5 |  | 686±43.8 |  |
| N2 | 119 | 582.6±57.7 |  | 745.4±71.2 |  |
| N3 | 77 | 592.2±72.1 |  | 809.5±75.9 |  |
| Indeterminate | 20 | 58±175.2 |  | 154.8±145.4 |  |
| **Pathologic M stage** |  |  | 0.732 |  | 0.475 |
| M0 | 903 | 455.4±22.7 |  | 715.5±28.4 |  |
| M1 | 21 | 364.3±141 |  | 590.7±173.8 |  |
| Indeterminate | 161 | 483.3±54.9 |  | 787.1±66.1 |  |
| **AJCC Stage** |  |  |  |  |  |
| Stage I | 181 | 526.6±50.6 | 0.058 | 759.5±69.1 | 0.808 |
| Stage II | 617 | 404.7±28.1 |  | 719.2±34.9 |  |
| Stage III | 247 | 538.4±40.9 |  | 732.8±48.4 |  |
| Stage IV | 19 | 429.6±145.6 |  | 603.5±186.3 |  |
| Indeterminate | 21 | 503.5±166.5 |  | 551.4±165 |  |

**Supplementary Table S2. Details of 19 databases used in meta-analysis of prognosis of the ten hub genes**

| **No.** | **Database accession** | **Release Year** | **Array Type** | **Sample Size** | **Endpoint** | | | | **Genes** | | | | | | | | | |
| --- | --- | --- | --- | --- | --- | --- | --- | --- | --- | --- | --- | --- | --- | --- | --- | --- | --- | --- |
|  |  |  |  |  | DFS | DSS | DMFS | OS | CCL19 | CCL21 | CCR4 | CCR5 | CNR2 | GPR183 | P2RY13 | PENK | PNOC | CCR2 |
| 1 | GSE1378 | 2004 | Arcturus 22k | 60 | √ | √ | √ | √ | √ | √ | √ | √ |  | √ | √ | √ | √ |  |
| 2 | GSE1379 | 2004 | Arcturus 22k | 60 | √ |  |  |  | √ | √ | √ | √ |  | √ | √ | √ | √ |  |
| 3 | GSE2034 | 2005 | HG-U133A | 286 |  |  | √ |  | √ | √ | √ | √ | √ | √ | √ | √ | √ |  |
| 4 | GSE3143 | 2005 | HG-U95A | 158 |  |  |  | √ | √ | √ |  |  | √ | √ |  | √ | √ |  |
| 5 | GSE3494-GPL96 | 2005 | HG-U133A | 236 |  | √ |  |  | √ | √ | √ | √ | √ | √ | √ | √ | √ |  |
| 6 | GSE2990 | 2006 | HG-U133A | 189 | √ |  | √ |  | √ | √ | √ | √ | √ | √ | √ | √ | √ |  |
| 7 | GSE1456-GPL96 | 2006 | HG-U133A | 159 | √ | √ |  | √ | √ | √ | √ | √ | √ | √ | √ | √ | √ |  |
| 8 | GSE4922-GPL96 | 2006 | HG-U133A | 249 | √ |  |  |  | √ | √ | √ | √ | √ | √ | √ | √ | √ |  |
| 9 | GSE6532-GPL570 | 2007 | HG-U133_Plus_2 | 87 | √ |  | √ |  | √ | √ | √ | √ | √ | √ | √ | √ | √ |  |
| 10 | GSE7390 | 2007 | HG-U133A | 198 | √ |  | √ | √ | √ | √ | √ | √ | √ | √ | √ | √ | √ |  |
| 11 | GSE7378 | 2007 | U133AAofAv2 | 54 | √ |  |  |  | √ | √ | √ | √ | √ | √ | √ | √ | √ |  |
| 12 | E-TABM-158 | 2008 | HG-U133A | 117 | √ | √ | √ | √ | √ | √ | √ | √ | √ | √ | √ | √ | √ |  |
| 13 | GSE11121 | 2008 | HG-U133A | 200 |  |  | √ |  | √ | √ | √ | √ | √ | √ | √ | √ | √ |  |
| 14 | GSE12093 | 2008 | HG-U133A | 136 |  |  | √ |  | √ | √ | √ | √ | √ | √ | √ | √ | √ |  |
| 15 | GSE9195 | 2008 | HG-U133_Plus_2 | 77 | √ |  | √ |  | √ | √ | √ | √ | √ | √ | √ | √ | √ |  |
| 16 | GSE7849 | 2008 | HG-U95A | 76 | √ |  |  |  | √ | √ |  |  | √ | √ |  | √ | √ |  |
| 17 | GSE9893 | 2008 | MLRG Human 21K V12.0 | 155 |  |  |  | √ |  |  | √ | √ | √ |  |  | √ | √ |  |
| 18 | GSE12276 | 2009 | HG-U133_Plus_2 | 204 | √ |  |  |  | √ | √ | √ | √ | √ | √ | √ | √ | √ |  |
| 19 | GSE19615 | 2010 | HG-U133_Plus_2 | 115 |  |  | √ |  | √ | √ | √ | √ | √ | √ | √ | √ | √ |  |

**Supplementary Table S3. Univariate and multivariate cox regression of OS and RFS in TCGA database**

|  | **RFS(Univariate analysis)** | | **RFS(Multivariate analysis)** | | **OS(Univariate analysis)** | | **OS(Multivariate analysis)** | |
| --- | --- | --- | --- | --- | --- | --- | --- | --- |
|  | Odd Ratios (95%CI) | p-value | Odd Ratios (95%CI) | p-value | Odd Ratios (95%CI) | p-value | Odd Ratios (95%CI) | p-value |
| <=55 vs ＞55 years old | 0.82 | 0.32 | 0.77 | 0.434 | 0.6 | 0.002 | 0.58 | 0.032 |
|  | (0.54-1.22) |  | (0.40-1.48) |  | (0.43-0.83) |  | (0.35-0.95) |  |
| ILC vs IDC | 0.94 | 0.811 | 0.86 | 0.631 | 0.83 | 0.426 | 0.99 | 0.965 |
|  | (0.56-1.58) |  | (0.46-1.61) |  | (0.53-1.31) |  | (0.58-1.69) |  |
| ER+ vs ER- | 0.61 | 0.026 | 0.55 | 0.086 | 0.72 | 0.074 | 0.72 | 0.26 |
|  | (0.39-0.94) |  | (0.28-1.09) |  | (0.5-1.03) |  | (0.41-1.27) |  |
| PR+ vs PR- | 0.61 | 0.02 | 0.63 | 0.151 | 0.74 | 0.085 | 0.71 | 0.193 |
|  | (0.41-0.93) |  | (0.33-1.18) |  | (0.53-1.04) |  | (0.42-1.19) |  |
| HER2+ vs HER2- | 0.82 | 0.538 | 0.59 | 0.116 | 1.32 | 0.201 | 1.13 | 0.603 |
|  | (0.43-1.55) |  | (0.30-1.14) |  | (0.86-2.02) |  | (0.72-1.77) |  |
| Perimenopause vs Premenopause | 0.61 | 0.509 | 1.06 | 0.942 | 0.37 | 0.331 | 0.6 | 0.62 |
|  | (0.14-2.61) |  | (0.24-4.55) |  | (0.05-2.75) |  | (0.08-4.53) |  |
| Postmenopause vs Premenopause | 1.16 | 0.55 | 1.1 | 0.802 | 2.16 | 0.002 | 1.68 | 0.12 |
|  | (0.71-1.88) |  | (0.53-2.27) |  | (1.31-3.54) |  | (0.87-3.25) |  |
| pT3&pT4 vs pT1&pT2 | 2.2 | <0.001 | 1.35 | 0.318 | 1.7 | 0.004 | 0.88 | 0.617 |
|  | (1.41-3.42) |  | (0.75-2.42) |  | (1.18-2.44) |  | (0.53-1.45) |  |
| pN1&pN2&pN3 vs pN0 | 2.8 | <0.001 | 2.22 | 0.007 | 2.24 | <0.001 | 1.77 | 0.012 |
|  | (1.76-4.45) |  | (1.25-3.96) |  | (1.57-3.19) |  | (1.13-2.76) |  |
| pM1 vs pM0 | 3.96 | 0.001 | 2.04 | 0.126 | 4.41 | <0.001 | 2.18 | 0.011 |
|  | (1.71-9.14) |  | (0.82-5.06) |  | (2.6-7.48) |  | (1.19-3.98) |  |
| Stage III&IV vs Stage I&II | 3.23 | <0.001 | 1.85 | 0.048 | 2.56 | <0.001 | 1.91 | 0.014 |
|  | (2.13-4.89) |  | (1.01-3.40) |  | (1.83-3.57) |  | (1.14-3.2) |  |
| High stromal score vs Low | 1.07 | 0.732 | 1.23 | 0.467 | 1.01 | 0.967 | 1.2 | 0.414 |
|  | (0.72-1.61) |  | (0.70-2.17) |  | (0.73-1.39) |  | (0.77-1.88) |  |
| High immune score vs Low | 0.66 | 0.043 | 0.41 | 0.006 | 0.7 | 0.031 | 0.45 | 0.002 |
|  | (0.44-0.99) |  | (0.22-0.77) |  | (0.51-0.97) |  | (0.27-0.74) |  |
| High ESTIMATE score vs Low | 0.89 | 0.588 | 1.65 | 0.195 | 0.92 | 0.611 | 1.78 | 0.061 |
|  | (0.60-1.34) |  | (0.77-3.51) |  | (0.66-1.27) |  | (0.97-3.26) |  |

|  | **Hazard Ratios (95%CI)** | **p-value** | **Hazard Ratios (95%CI)** | **p-value** |
| --- | --- | --- | --- | --- |
| <=55 vs ＞55 years old | 0.52 | <0.001 | 0.44 | <0.001 |
|  | (0.45-0.60) |  | (0.35-0.57) |  |
| ILC vs IDC | 1.01 | 0.954 | 1.07 | 0.568 |
|  | (0.80-1.26) |  | (0.85-1.34) |  |
| ER+ vs ER- | 0.8477 | 0.022 | 0.85 | 0.082 |
|  | (0.74-0.98) |  | (0.71-1.02) |  |
| PR+ vs PR- | 0.79 | <0.001 | 0.91 | 0.177 |
|  | (0.70-0.89) |  | (0.78-1.04) |  |
| HER2+ vs HER2- | 1.45 | <0.001 | 1.44 | <0.001 |
|  | (1.22-1.73) |  | (1.20-1.73) |  |
| Tumor Size>2cm vs <=2cm | 1.74 | <0.001 | 1.51 | <0.001 |
|  | (1.53-1.96) |  | (1.33-1.71) |  |
| LN+ vs LN- | 1.67 | <0.001 | 1.59 | <0.001 |
|  | (1.48-1.88) |  | (1.33-1.90) |  |
| Grade III vs Grade I&II | 1.33 | <0.001 | 1.13 | 0.169 |
|  | (1.18-1.50) |  | (0.95-1.33) |  |
| High immune score vs Low | 0.91 | 0.106 | 0.88 | 0.039 |
|  | (0.80-1.02) |  | (0.77-0.99) |  |

**Supplementary Table S4. Univariate and multivariate cox regression of OS in METABRIC database**

Supplementary Figure Legends

Supplementary Figure S1. Meta-analysis of genes prognosis as (A)-(I) shows CCL19, CCL21, CCR4, CCR5, CNR2, GPR183, P2RY13, PENK and PNOC. Diamonds indicated the result of meta-analysis of HR,

Supplementary Figure S2. Kaplan Meier analyses of genes in TCGA and METABRIC if they are not significantly related to the prognosis of patients.

Supplementary Figure S3. Scatter diagrams of hub genes’ expression and tumor purity, infiltrated immune cells(B cells, CD8+ T Cell, CD4+T Cell, macrophages, neutrophils and dendritic cells) from TIMER web tool as stated in Results. Partial correlation coefficients and p-values are sketched in the graph.

Supplementary Figure S4. (A)&(B) showed the linear relativity between stromal score/ESTIMATE score and immune score in TCGA database. They appeared to have similar trend as in the relationship in METABRIC (C)&(D).
