# Supplementary figures and images for: Immune and Stroma Related Genes in Breast Cancer: A Comprehensive Analysis of Tumor Microenvironment Based on the Cancer Genome Atlas (TCGA) Database
Source: Front Med (Lausanne). 2020 Mar 5;7:64. doi: 10.3389/fmed.2020.00064 (PMC7066229; doi:10.3389/fmed.2020.00064)

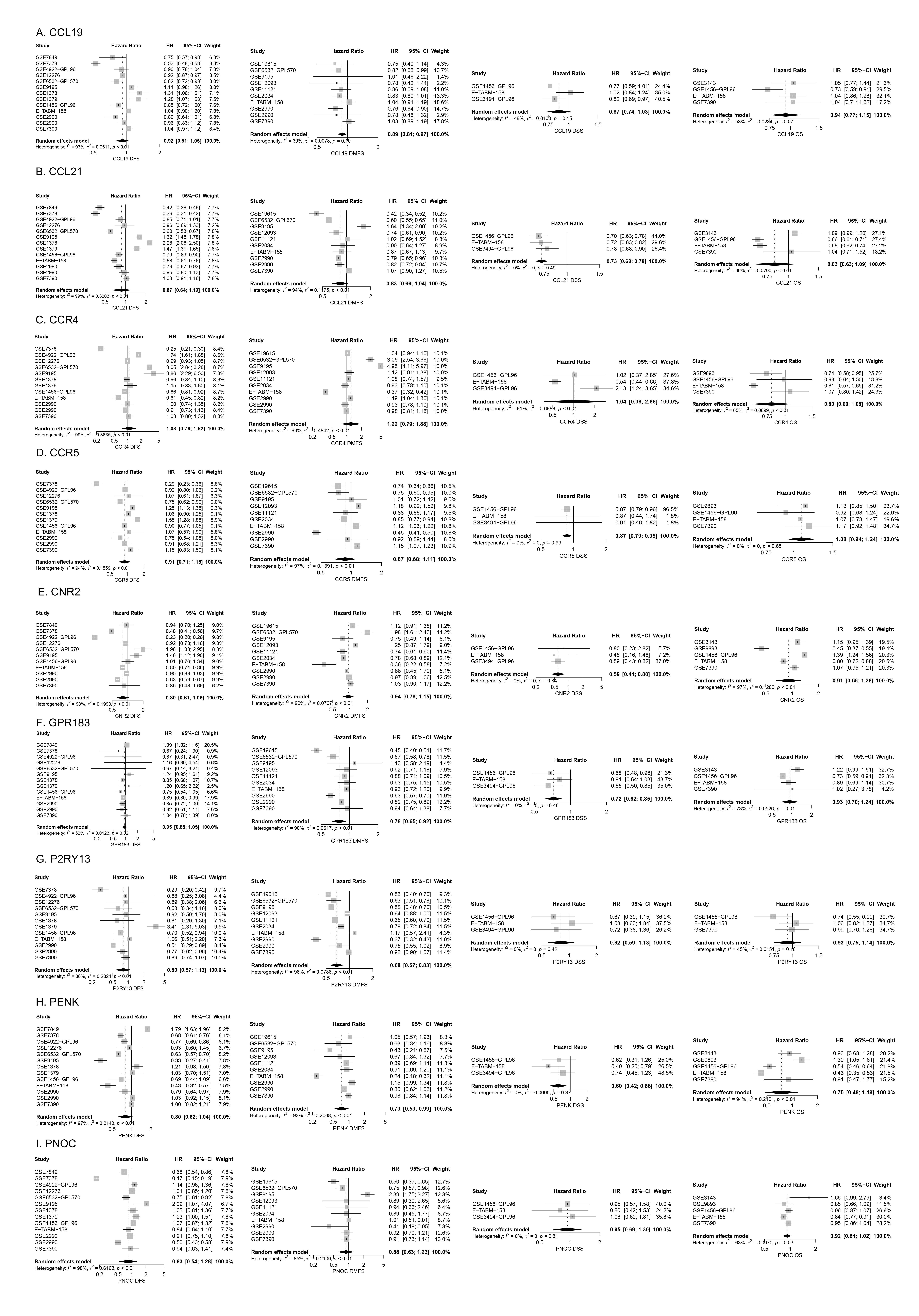

Supplement: Supplementary file 3 [file Image_1.TIF]

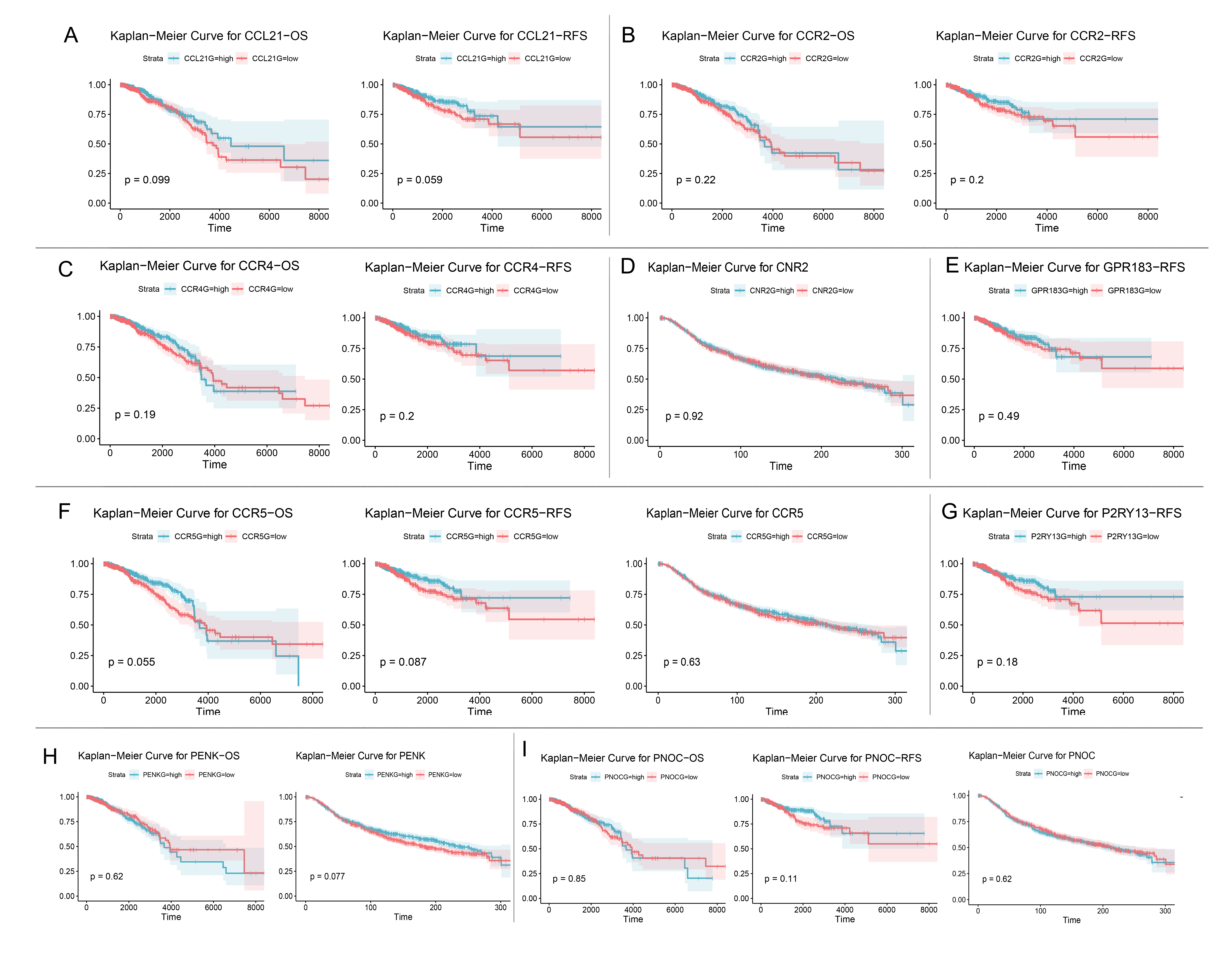

Supplement: Supplementary file 4 [file Image_2.TIF]

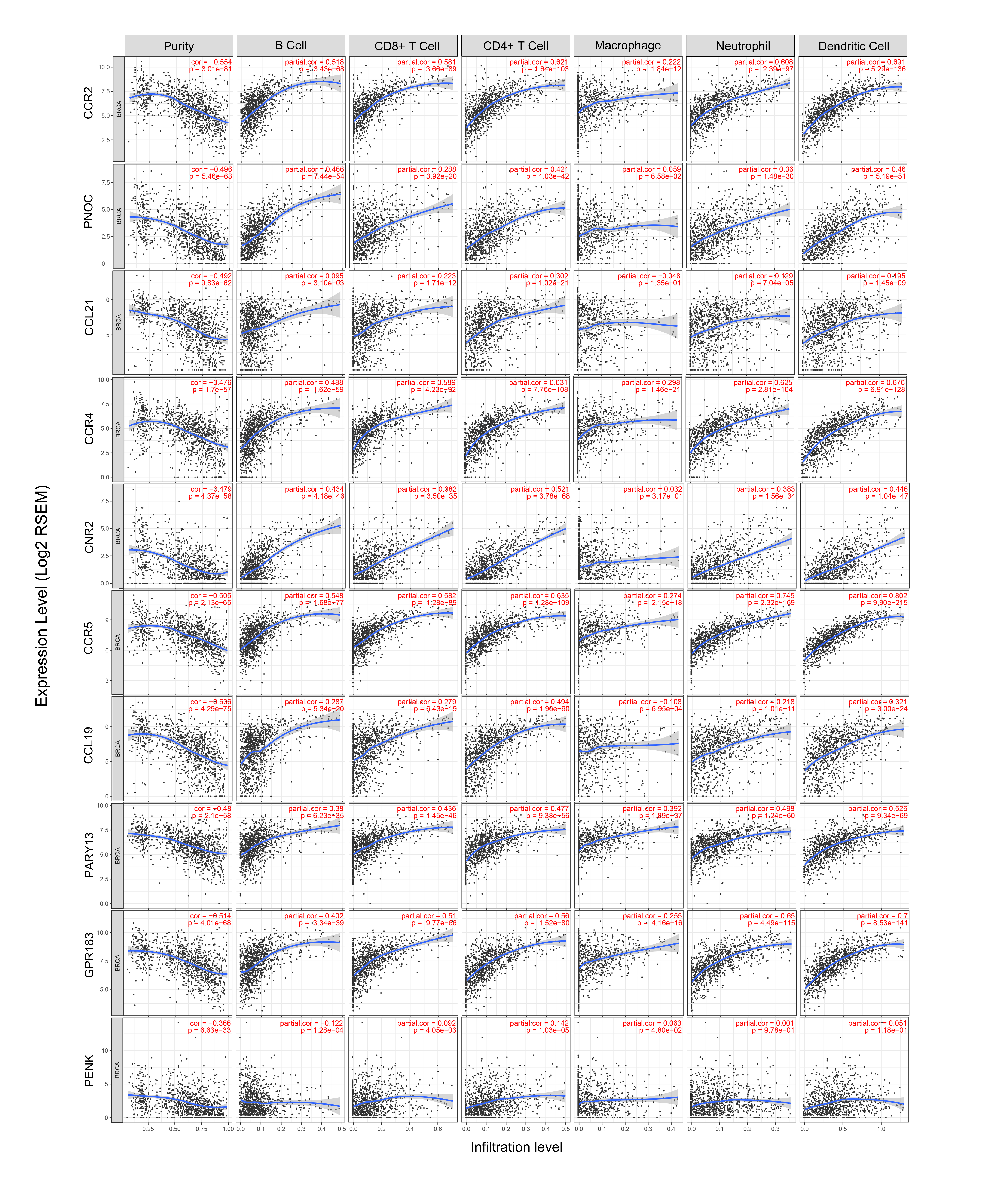

Supplement: Supplementary file 5 [file Image_3.TIF]

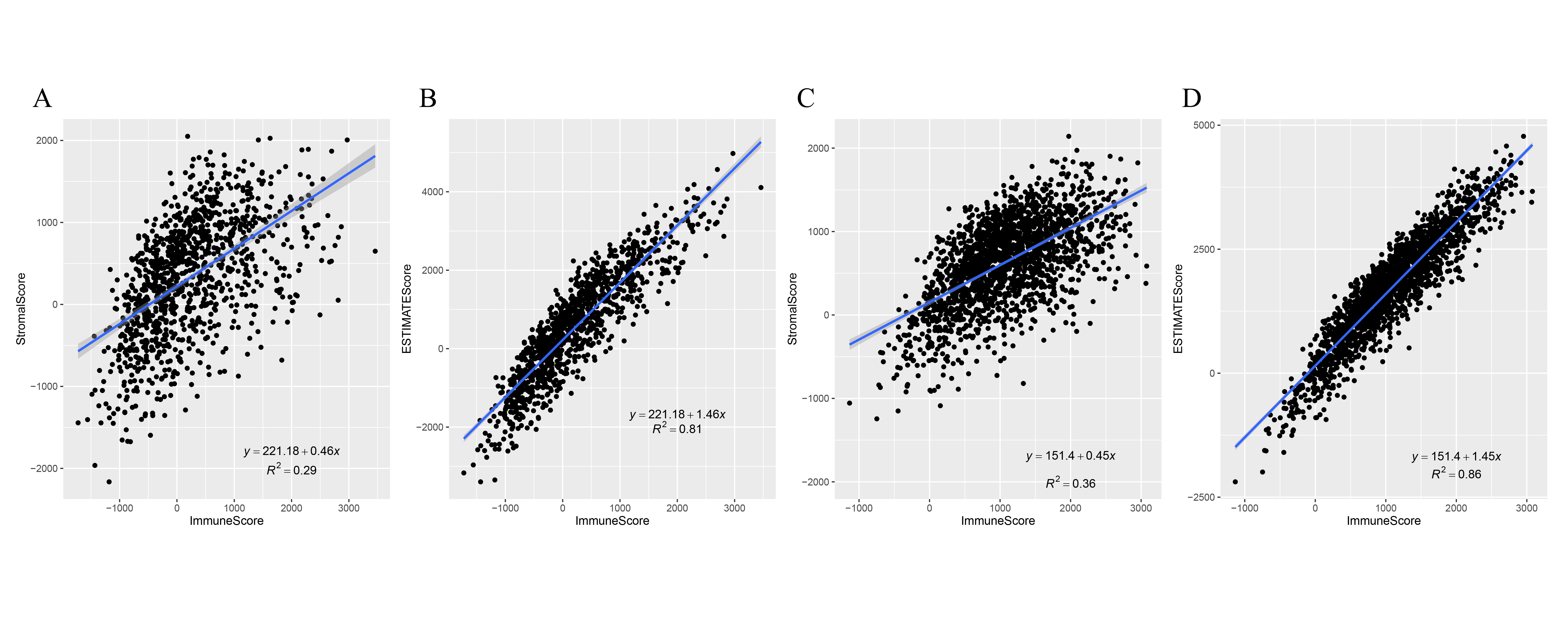

Supplement: Supplementary file 6 [file Image_4.TIF]
